# Supplementary material for: Hydropower capacity factors trending down in the United States
Source: Nat Commun. 2024 Jun 27;15:5445. doi: 10.1038/s41467-024-49553-x (PMC11211397; doi:10.1038/s41467-024-49553-x)
Supplement: Supplementary file 1 — Supplementary Information [file 41467_2024_49553_MOESM1_ESM.pdf]

Supplementary Information for
Hydropower Capacity Factors Trending Down in the United States

Sean W. D. Turner, Ganesh R. Ghimire, Carly Hansen, Debjani Singh, Shih-Chieh Kao

Sean Turner
Email: turnersw@ornl.gov

This PDF file includes:

Figure S1 – Robustness of long-term trend to analysis period.

Figure S2 – Trends in annual flow maxima and seasonality at plants for which change in annual water availability cannot explain CF decline.

Figure S3 – Trends in annual flow maxima and seasonality at plants for which change in annual water availability cannot explain CF decline.

Figure S4 – CF trend along dimensions of plant age, FERC-license status, and mode of operation.

Figure S5 – Isolated cases with apparent impact of capacity upgrade on CF downshift.

Figure S6 – Graphical depiction of the Capacity Factor model under alternative parameter settings.

Figure S7 – Examples of CF model on a run-of-river and storage plant.

Figure S8 – Cross-validation of the CF model, compared to the linear model.

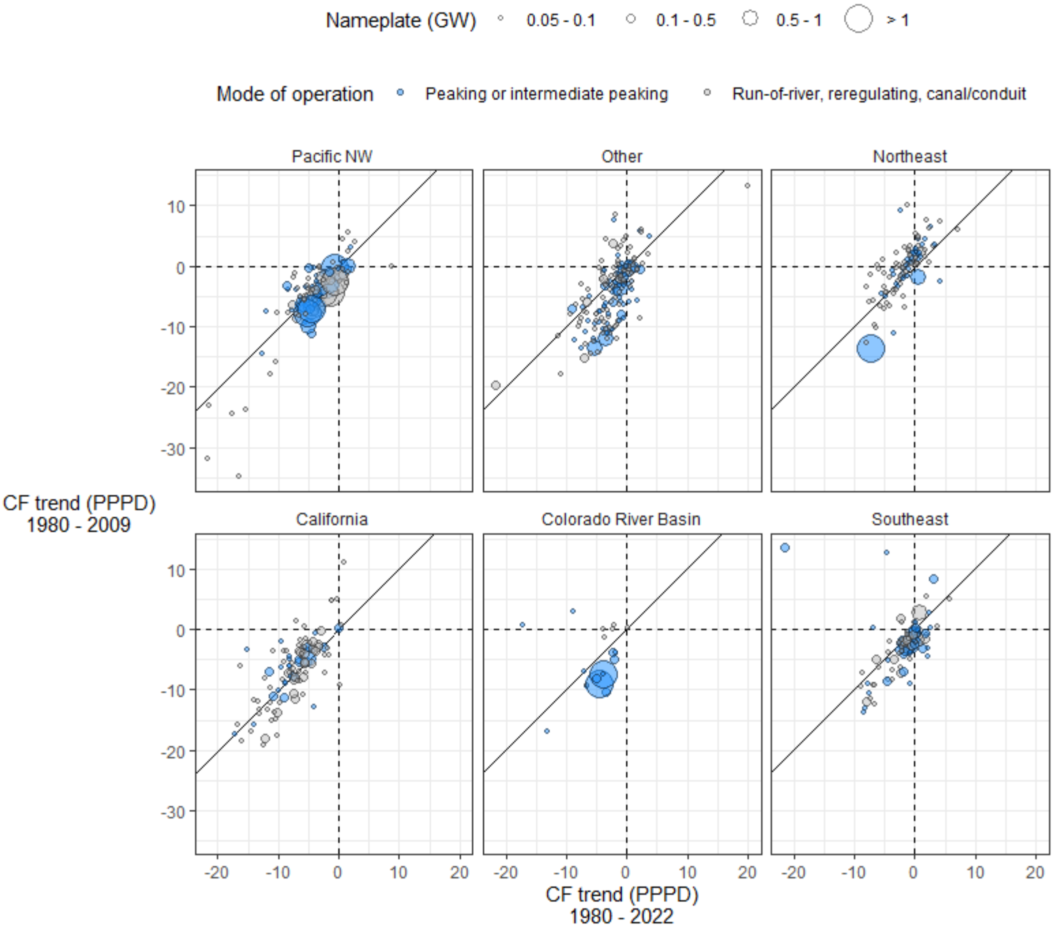

Figure S1 – Comparison of capacity factor (CF) trends computed using 1980 – 2022 versus the 30-year period 1980 – 2009. Each point represents CF trend at one of 610 hydropower plants with >5MW nameplate. Trends analyzed for the 30-year period 1980-2009 exclude effects of recent droughts in California as well as possible effects of renewables integration.

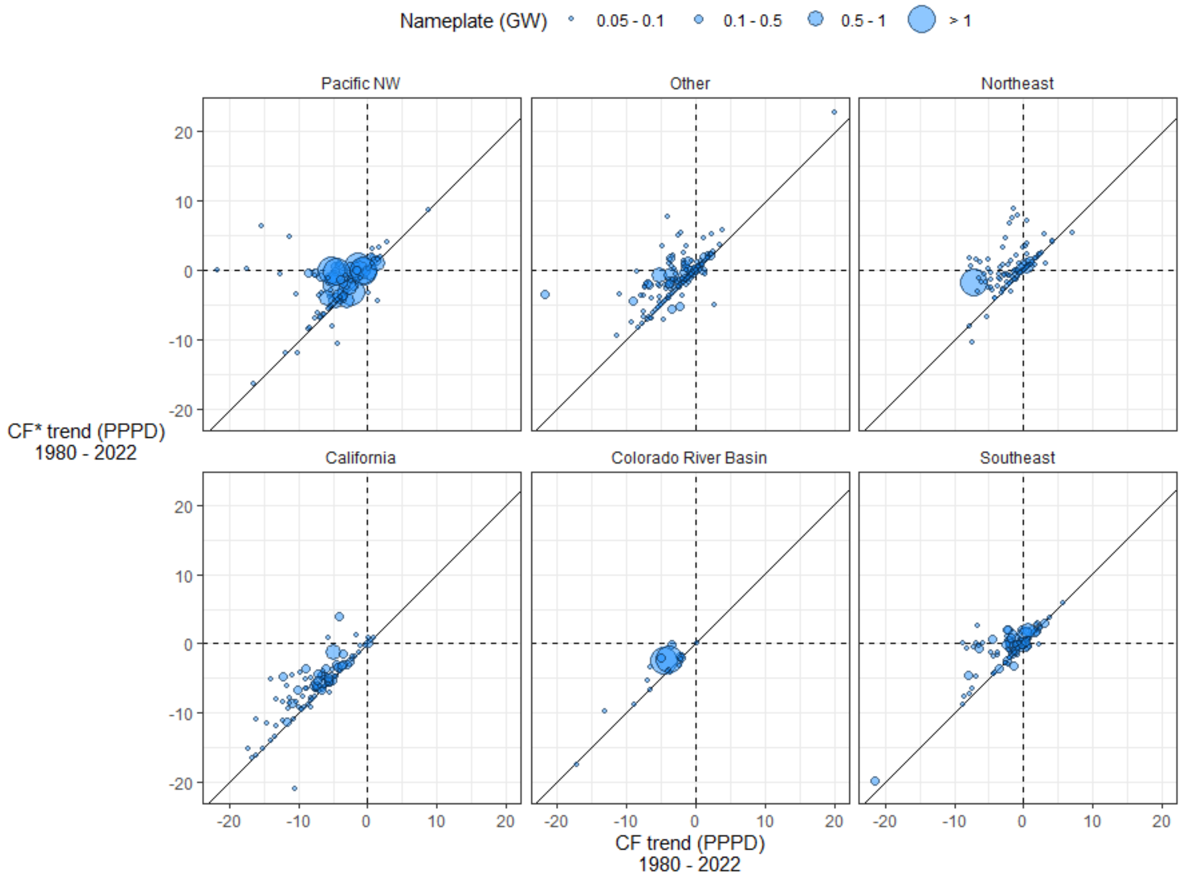

Figure S2 – Regional trend comparison, CF versus CF recomputed with a fixed nameplate (equivalent to showing actual generation trend). The comparison highlights the impacts of capacity additions (above the 45-degree line) and retirements (below the 45-degree line) on results.

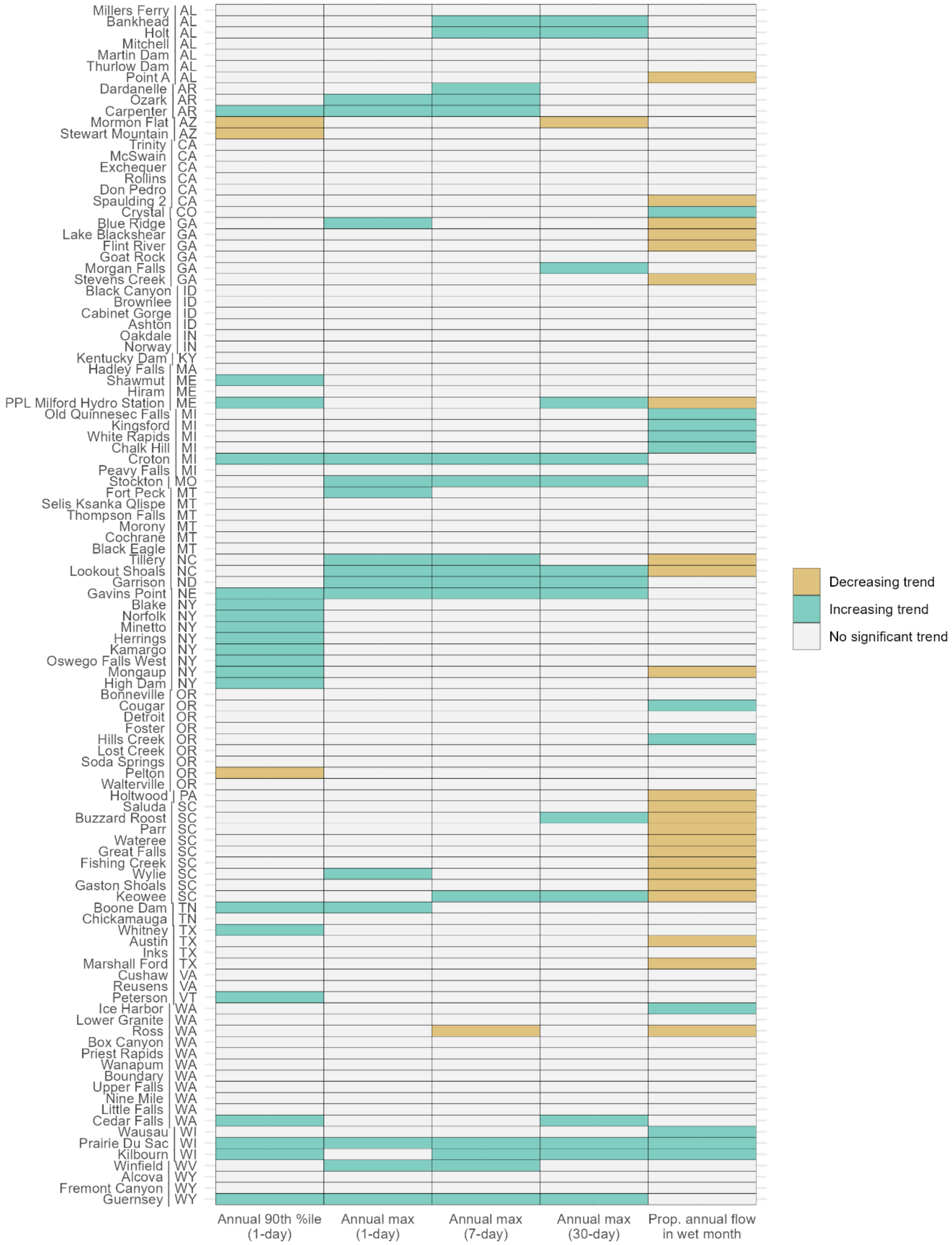

Figure S3 – Analysis of trend in hydrological extremes (90th percentile of daily flow and 1-day, 7-day, and 30-day annual maxima) and seasonality of hydrology (proportion of annual flow in wettest month). Trends analyzed for 107 plants that show CF decline are not well explained by changes in annual water availability.

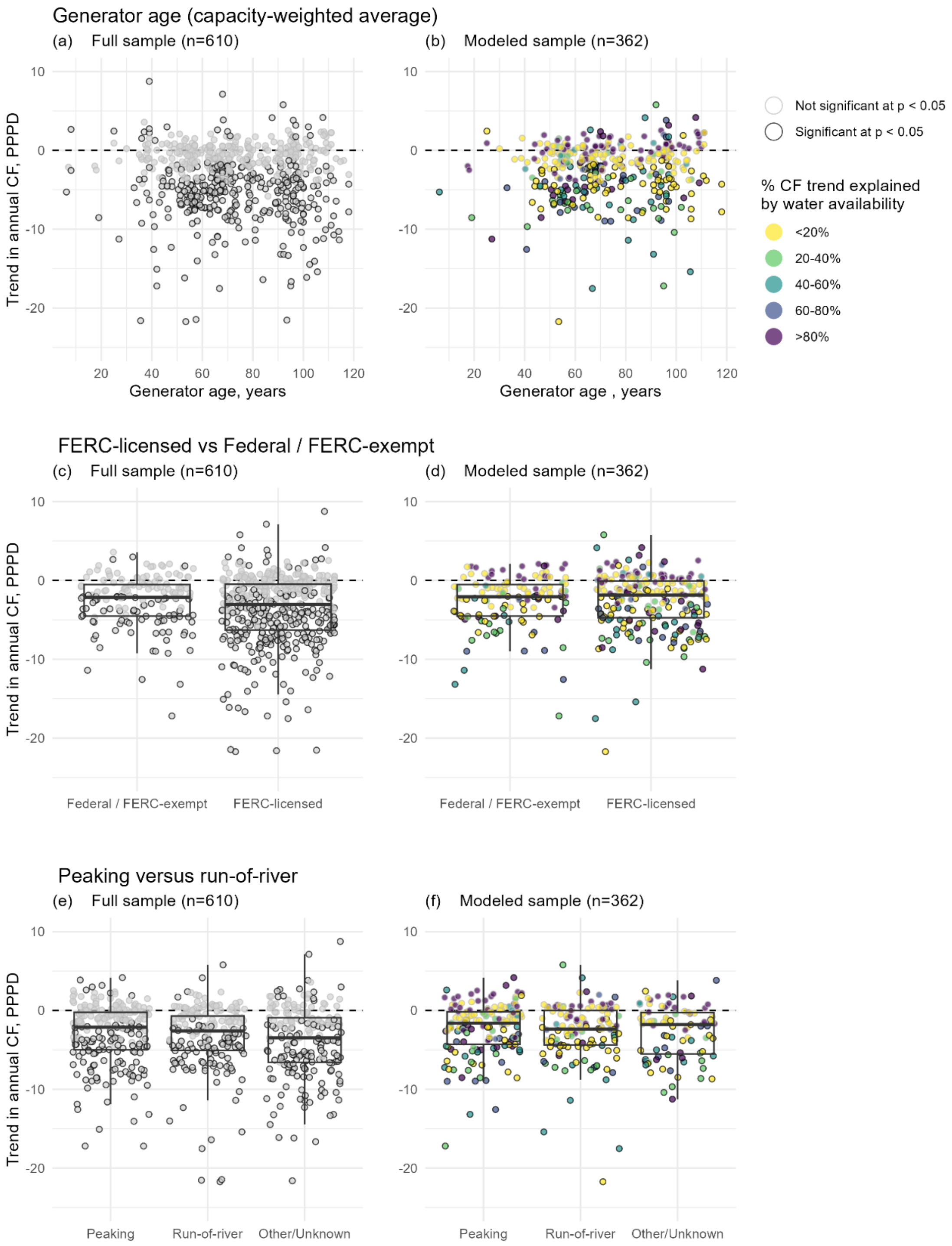

Figure S4 – CF trend for all plants (left-hand side) and modeled plants (right-hand side) along dimensions of generator age (capacity weighted mean age across units at each plant) in panels a and b, FERC-license status in panels c and d, and mode of operation in panels e and f.

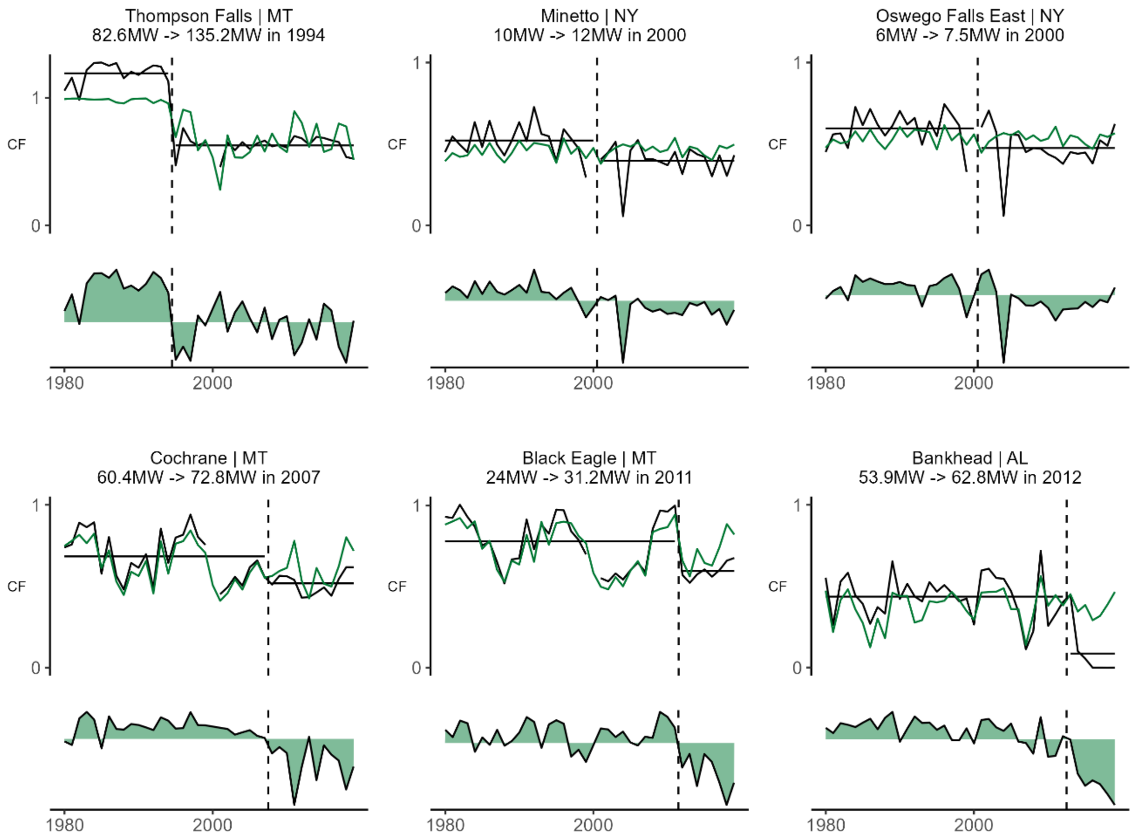

Figure S5 – Small number of cases found for which a downshift in CF (measured by the residual between modeled and observed CF) coincides with capacity upgrades at plants. Green line represents modeled CF (with water availability) while the lower series in each graphic gives the residual (observed minus modeled CF).

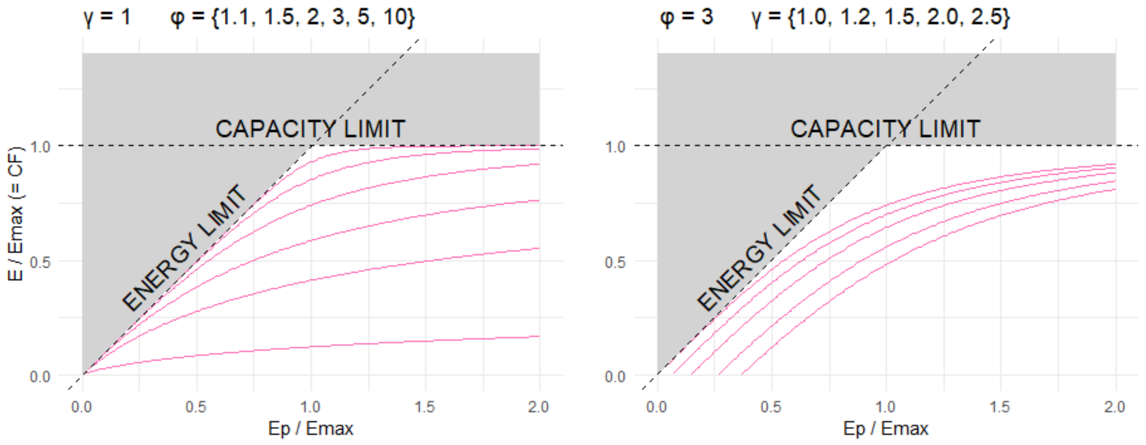

Figure S6 – Graphical depiction of the capacity factor model based on capacity potential. The left-hand side is equivalent to the one-parameter model, which can capture a wide range of conditions but must pass through the origin. The right-hand side shows the influence of the second parameter, which allows one to shift the CF model to the right.

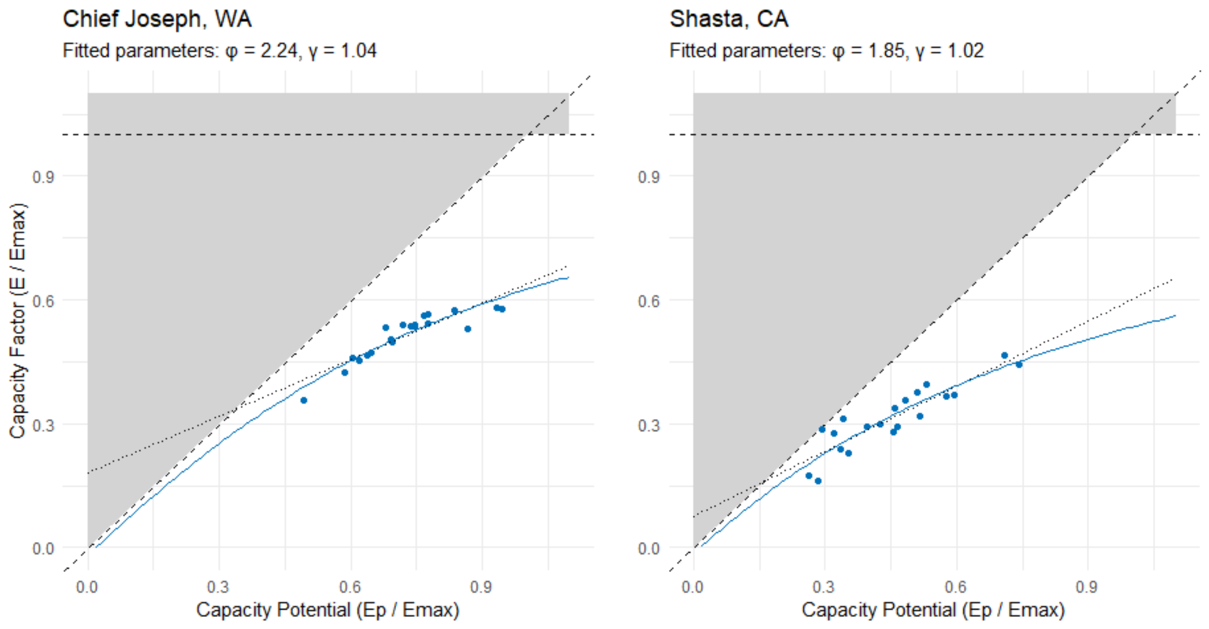

Figure S7 – Two examples of a two-parameter CF model based on available water (represented as capacity potential), with each point representing a year of data (2000-2019). Both examples also show the corresponding linear model, which overestimates CF at both high and low CF. The linear model also strays into non-feasible CF values when extrapolated to very low levels of potential (i.e., very dry years).

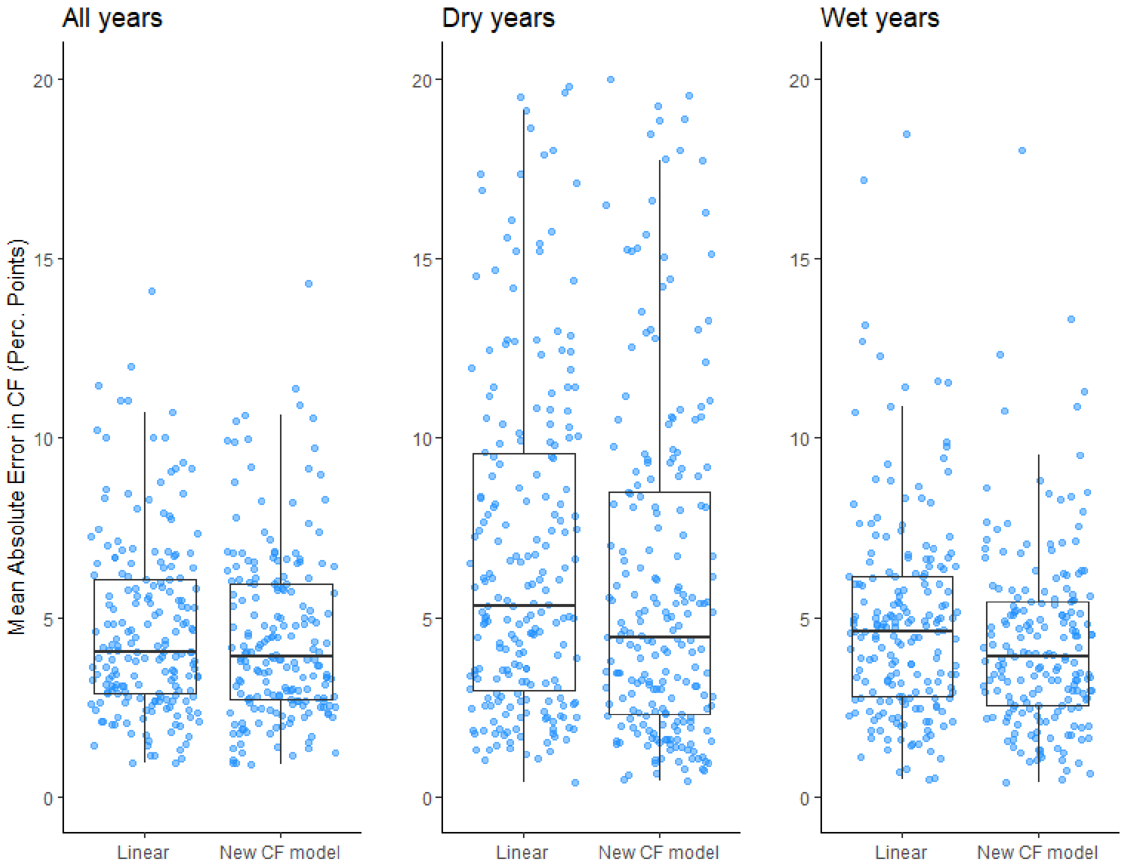

Figure S8 – Leave-one-out cross-validation of CF model, compared against a linear model predicting CF from potential energy (water availability) at each plant based on years 2000 – 2019 (to avoid significant operational change). Each year's result for each dam is based on the model trained with all data except for that year. The resulting error is averaged for all years (left-hand panel), the driest four years (middle panel), and the wettest four years (right-hand panel). Each point represents a different hydropower plant.
